# Overexpression of Long Noncoding RNA H19 Downregulates miR-140-5p and Activates PI3K/AKT Signaling Pathway to Promote Invasion, Migration and Epithelial-Mesenchymal Transition of Ovarian Cancer Cells

**DOI:** 10.1155/2021/6619730

**Published:** 2021-06-21

**Authors:** Hao Xu, Yuan Ding, Xiangying Yang

**Affiliations:** ^1^Department of Traditional Chinese Medicine, Affiliated Hangzhou First People's Hospital, Zhejiang University School of Medicine, Hangzhou, Zhejiang Province, China; ^2^ICU Affiliated Hangzhou First People's Hospital, Zhejiang University School of Medicine, Hangzhou, Zhejiang Province, China

## Abstract

**Objective:**

The abnormal expression of LncRNA H19 and miR-140-5p has been linked to ovarian cancer (OC). Whether H19 directly regulates miR-140-5p in ovarian cancer cells has been unclear. In this study, we deeply explored the relationship between H19 and miR-140-5p in ovarian cancer and the mechanism of action in regulating OC progression.

**Methods:**

A total of 66 patients with OC admitted to the hospital from June 2017 to June 2019 were selected as the research group (RG), and meanwhile, 60 cases of healthy subjects were selected as the control group (CG). In addition, OC cells and normal ovarian epithelial cells were used to detect H19 and miR-140-5p expression levels and to analyze the effect of H19 on OC cells. The activation of the PI3K/AKT pathway and downstream proteins were analyzed by western blot.

**Results:**

H19 was highly expressed while miR-140-5p was lowly expressed in OC patients and cell lines (*P* < 0.050). The proliferation, invasion, migration ability, and epithelial-mesenchymal transition (EMT) of OC cells were reduced after inhibiting H19 expression, and the apoptosis rate was increased. Transfection of cells with miR-140-5p mimics brought opposite effects. Online prediction and dual-luciferase reporter (DLR) confirmed that H19 directly binds miR-140-5p. Western blot assay indicated overexpression activated the PI3K/AKT signaling pathway in OC cells. Moreover, overexpression promoted tumor growth in nude mice and was suppressed by PI3K inhibitor.

**Conclusion:**

LncRNA H19 downregulation of miR-140-5p to activate the PI3K/AKT signaling pathway and promote the proliferation, invasion, migration and EMT of OC.

## 1. Introduction

Ovarian cancer (OC) is currently one of the most frequently occurring tumors worldwide, accounting for approximately 4% of all female systemic malignancies [1]. It is estimated that the number of new OC patients worldwide has exceeded 300000 in 2016, which is about six times higher than that of a decade ago [2, 3]. In addition, surveys have shown that there is an increasing incidence of OC at younger ages in recent years [3]. Moreover, at present, the fatality rate of OC is the highest among female malignant tumors, and the 5-year mortality rate of patients with advanced prognosis is as high as 63.03% [1].

LncRNA, as a highly active RNA, has been proved to participate in the genesis and development of a number of tumors [4]. Among them, lncRNA H19 has been found to be abnormally expressed in human malignant tumors and regulates cell proliferation, migration, invasion, antiapoptosis, and epithelial-mesenchymal transition (EMT) through various mechanisms, thus playing a carcinogenic or anticancer role [5–7]. H19 has been found to act as a microRNA sponge to indirectly regulate the expression of microRNA downstream target genes thus mediating cancer progression in several cancer types [8–10]. Even in the same cancer, H19 also sponges various microRNAs to mediate diverse regulatory mechanisms [11, 12]. Overexpression of H19 has been linked to the cisplatin resistance and migration of ovarian cancer during EMT [13, 14]. It acted as a competing endogenous RNA of miR-370-3p to promote EMT of ovarian cancer cells [15]. Moreover, H19 promotes small cell lung cancer (SCLC) progression via sponging miR-140-5p [16]. Besides, in regulating SCLC, overexpression of miR-140-5p has been shown to restrict the proliferation, migration, and invasion abilities and accelerated cell apoptosis in ovarian cancer cell lines [17, 18].

Although the abnormal expression of LncRNA H19 and miR-140-5p has been linked to ovarian cancer, whether H19 directly regulates miR-140-5p in ovarian cancer cells has been unclear. In this study, we deeply explored the relationship between H19 and miR-140-5p in ovarian cancer and the mechanism of action in regulating OC progression. We found that overexpression of H19 in ovarian cancer cells downregulated miR-140-5p and promoted invasion, migration, and epithelial-mesenchymal transition of ovarian cancer cells by the activation of the PI3K/AKT signaling pathway. Our study provided a new reference for the future clinical diagnosis and treatment of OC.

## 2. Materials and Methods

### 2.1. General Information

A prospective analysis was performed on 66 patients with OC (research group, hereinafter referred to as RG) who were admitted to the hospital from June 2017 to June 2019 and 60 healthy subjects (control group, hereinafter referred to as CG) who underwent physical examinations during the same period. No significant differences were noticed in clinical baseline data such as age and BMI between the two groups (*P* > 0.050). This experiment had been approved by the Ethics Committee of Affiliated Hangzhou First People's Hospital, Zhejiang University School of Medicine, and all the above subjects had signed the informed consent.

### 2.2. Inclusion and Exclusion Criteria

Inclusion criteria: patients aged 30-60 years old, who were diagnosed with OC by pathology for the first time. Exclusion criteria: patients who had received chemoradiotherapy; patients with other malignant tumors; patients with severe liver and kidney dysfunction; patients with severe infectious diseases; patients with expected survival of less than 1 month; patients with poor treatment compliance for mental disorders; patients transferred from other hospitals.

### 2.3. Blood Sample Collection

Fasting venous blood (4 mL) was extracted from the two groups in the morning, placed at room temperature for 30 min, and centrifuged for 10 min (400 × g). Then, the obtained upper serum was preserved in the refrigerator at -80°C for testing.

### 2.4. Cell Data

OC cells A2780, HO-8910, Anglne, NIH : OVCAR-3, and normal ovarian epithelial cells IOSE80 were all purchased from ATCC. 10% fetal bovine serum was added to the MEM medium, while the DMEM high glucose medium was blended with 10% fetal bovine serum, and the culture was carried out in an environment containing 5%CO_2_ and 37°C.

### 2.5. Cell Transfection

When the cells converged to approximately 80%, they were digested with 0.25% trypsin and adjusted to 4 × 10^8^/L with the help of a complete culture medium. Then, 100 *μ*L of cell culture solution was inoculated into new 6-well plates and placed in a CO_2_ incubator for 24 h. After that, the overexpression sequence sh, mimics of H19 and miR-140-5p and the inhibitory sequence si, and inhibition of H19 and miR-140-5p were transfected into the cells by ExFect 2000 Transfection Reagent, and the blank group (NC) was set.

### 2.6. PCR Detection

Two groups of serum and adherent cells (prepared as cell suspension) were obtained, and 1 mL Trizol was added to extract total RNA. The total RNAOD value at 260-280 nm was measured by ultraviolet spectrophotometer, and those with RNAOD>1.8 were used for subsequent qPCR quantification. FastQuant RT Super Mix (KR108) kit (Tiangen Biotechnology Co., Ltd., Beijing, China) was in charge of reverse transcription. The reaction system and procedures of reverse transcription were as follows: 5 × FQ − RT Super Mix: 5 *μ*L, total RNA: 1 *μ*g, and replenished the system to 20 *μ*L with RNase-Free ddH2O; reverse transcription reaction: 42°C/15 min, enzyme extinguishing: 95°C/3 min. CDNA was collected for PCR amplification. The primer sequences are shown in Table 1. QPCR amplification system: cDNA: 1 *μ*L, upstream and downstream primers: 0.4 *μ*L each, 2 × TransTaq ® Tip Green qPCR SuperMix: 10 *μ*L, Passive Reference Dye (50x): 0.4 *μ*L, and finally added to ddH2O complete to 20 *μ*L. QPCR amplification conditions: predenaturation: 94°C/30 s, denaturation: 94°C/5 s, annealing and extension: 60°C/30 s, totalling 40 cycles. Each sample was equipped with three replicate wells, and the experiment was conducted for a total of three times. GADPH was used as the internal reference for H19, U6 as the internal reference for miR-140-5p, and the data was analyzed by 2^-*△△*ct^.

### 2.7. MTT

Four 96-well plates were selected, and three wells were randomly selected from each well plate to inoculate cells, ensuring a cell density of 5 × 10^3^/100 *μ*L per well. A well plate was taken out 24, 48, 72, and 96 h after inoculation, at which time points 5 mg/ml MTT solution (MTT dissolved in DMSO, Solarbio) at 10 *μ*L/well was added before it resumed to culture for 1 h. The culture medium was then discarded, and the OD value at 570 nm was tested with the help of a microplate reader.

### 2.8. Flow Cytometry

After transfection, the cells were processed for digestion with 0.25% trypsin, then washed with PBS twice, and mixed with 100 *μ*L binding buffer to prepare a suspension of 1 × 10^6^ cells/mL. Followed by the successive addition of Annexin V-FITC and PI before incubating at room temperature for 5 min away from light. Finally, the test was conducted using the CytoFLE S flow cytometry system. The experiment was repeated three times to average the value.

### 2.9. Transwell

Cells with a specification of 2 × 10^4^ were inoculated in the upper Transwell chamber (200 *μ*L mixed solution containing 10% fetal bovine serum and 1%DMEM medium), and the lower one was added with the DMEM medium (containing 10% FBS with a total volume of 500 *μ*L). The Transwell chamber was placed at 37°C and cultured in 5%CO_2_ for 24 h, after which the upper compartment liquid was removed and the cell wall was erased. The cells on the opposite side of the Transwell chamber were immobilized with 4% poly methanol for 20 min, followed by a 15-minute staining with crystal violet, and then cleaned with PBS buffer solution. Photos of cell migration were taken under a 200-fold microscope, and the cell number was counted in 3 randomly selected fields of view. The average value was taken as the number of transmembrane cells. The experiment was repeated three times. Saving that 8% matrix glue was padded, and the number of cells per well was increased to 5 × 10^4^, the steps of cell invasion were the same as those of cell invasion as mentioned above.

### 2.10. Wound-Healing Assay

In 6-well plates, the transfected cells were grown at a density of approximately 2 × 10^5^ cells per well. After 24 hours, a wound was created through the diameter of the 6-well plate with a small pipette tip. The floating cells were then gently washed off with PBS, and 5 fields were randomly selected from each well for observation under a 20-fold microscope. Then, the cells were cultured in a serum-free medium and observed at 3 fields 24 and 72 hours later.

### 2.11. Western Blot

Protein extract, which was 20 mM Tris-HCl solution (pH 7.5, Solarbio company) mixed with protein inhibitor (Solarbio company), was employed to process the cell protein. The adherent cells were prepared as a cell suspension, then added with 1 mL of cell protein extract before it was repeatedly pipetted until the cells were completely lysed. Then, the extract was centrifuged in a precooled centrifuge at 4°C for 20 min, 1.6104 g. Next, the supernatant was taken, whose concentration was tested by BCA. The protein was then isolated by SDS-PAGE electrophoresis before transferring it to the NC membrane and left at room temperature for 1 h (closed with 5% skimmed milk-PBS solution). The primary antibodies were then added and left to stand overnight at 4°C. After that, the NC membrane was washed with PBS solution for three times, followed by the addition of goat anti-rabbit secondary antibody (HRP cross-linking), and then placed at indoor temperature for 1 h. Finally, the NC membrane was washed with PBS solution and visualized using ECL luminescent solution. GAPDH was used as the internal reference protein, and the relative expression level of the protein to be measured was equal to the gray value of the band to be measured/the gray value of the GAPDH band. PI3K, E-cadherin, N-cadherin, AKT, p-AKT (Thr308), p-AKT (Ser473), vimentin primary antibody, and secondary antibody goat anti-rabbit (HRP cross-linked) were all purchased from Abcam. Primary antibodies for p-mTOR (Ser2448), p-FoxO1 (Ser256), p-MDM2 (Ser166), p-NF-*κ*B p65 (Ser536), p-GSK-3*β* (Ser9), PTEN, GSK-3*β*, mTOR, FoxO1, MDM2, and NF-*κ*B p65 were from Santa Cruz.

### 2.12. DLR Assay

The complementary DNA fragment containing wild-type (H19-WT) or mutant H19 (H19-mut) fragments was subcloned to the downstream of the luciferase gene in the psi-CHECK2 luciferase reporter vector. Transfection reagents from Invitrogen, USA, were adapted to cotransfect miR-140-5p mimics or miR-140-5p inhibitors with H19-WT or H19-mut reporter vectors. Forty-eight hours after transfection, the luciferase activity of firefly and renin in cell lysates was continuously measured using a DLR kit (Promega, USA).

### 2.13. Tumorigenesis Experiment in Nude Mice

Female BALB/c nude mice, aged 5-week, were raised in a sterile environment, and then, the 3 × 10^6^ A2780 cells transfected with stable H19-sh and its control plasmid were suspended in 100 *μ*L phosphate buffer and subcutaneously injected into the dorsal subcutaneous of nude mice. For PI3K inhibition, mice were orally administrated with 50 mg/kg XL-765 (Selleck) once per day [19]. The tumor growth of 5 nude mice in each group was detected every 7 days and worked out by the formula of volume = length^∗^ width^2∗^0.5^2^. Twenty-eight days after injection, the mice were euthanized and the size and mass of the tumor were accurately measured. The animal study was reviewed and approved by Affiliated Hangzhou First People's Hospital, Zhejiang University School of Medicine.

### 2.14. Coimmunoprecipitation

Ovarian cancer cells were lysed using a RIP kit (Millipore, Billerica, MA, USA) and incubated with protein A magnetic beads, which were conjugated with antibodies at 4°C. After 6 hours, the beads were washed with washing buffer and, then, incubated with 0.1% SDS/0.5 mg/ml proteinase K at 55°C for 30 minutes to remove proteins and subjected to qRT-PCR analysis.

### 2.15. Statistical Methods

The results of this experiment were statistically analyzed by SPSS 24.0 (Yuchuang Network Technology Co., Ltd., Shanghai, China), and all graphical results were plotted using Graphpad8 (Softhead Technology Co., Ltd., Shenzhen, China). All the experimental results were described in the form of (mean ± standard deviation). *T*-test was employed for intergroup comparisons, one-way ANOVA and LSD posttest were applied for multigroup comparisons, and repeated measurement analysis of variance and Bonferroni posttest were adopted for multipoint comparisons. The diagnostic and predictive value was analyzed by the ROC curve, and the correlation was analyzed via Pearson correlation coefficient. *P* < 0.050 indicated a statistically significant difference.

## 3. Results

### 3.1. Clinical Significance and Relationship between H19 and miR-140-5p in OC

**Figure 1:**
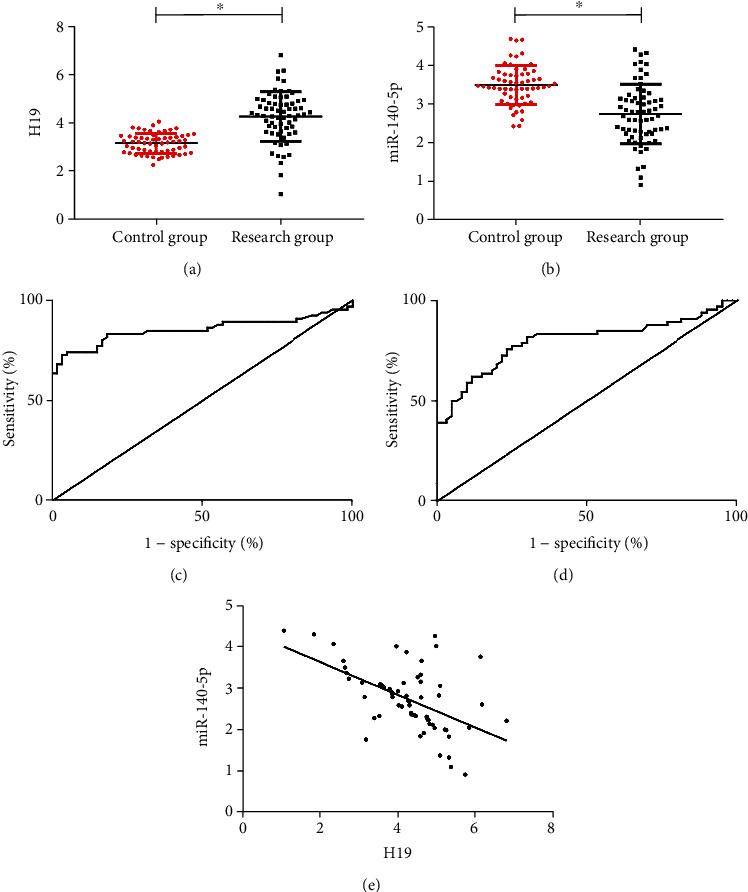
Clinical significance and relationship between H19 and miR-140-5p in OC. (a) Comparison of serum H19 expression level between the RG and the CG, ^∗^ indicated *P* < 0.050. (b) Comparison of serum miR-140-5p level between the RG and the CG, ^∗^ indicated *P* < 0.050. (c) ROC curve of H19 for predicting the occurrence of OC. (d) ROC curve of miR-140-5p for predicting OC. (e) Correlation between H19 and miR-140-5p in the RG.

Compared with the healthy control group, the serum expression level of H19 was markedly higher (*P* < 0.050) (Figure 1(a)), while the serum miR-140-5p level was notably lower in the patients with OC (research group) (*P* < 0.050) (Figure 1(b)). According to ROC curve analysis, when the cut-off value was greater than 3.825, the diagnostic sensitivity of H19 to predict the occurrence of OC was 72.73% and the specificity was 96.67% (*P* < 0.050) (Figure 1(c)). The sensitivity and specificity of miR-140-5p in predicting the occurrence of OC were 75.76% and 76.67% when the cut-off value was less than 3.145 (*P* < 0.001) (Figure 1(d)). Pearson correlation coefficient analysis revealed that H19 was negatively linked with miR-140-5p (*r* = −0.529, *P* < 0.001) (Figure 1(e)).

### 3.2. Effects of H19 on OC Cells

**Figure 2:**
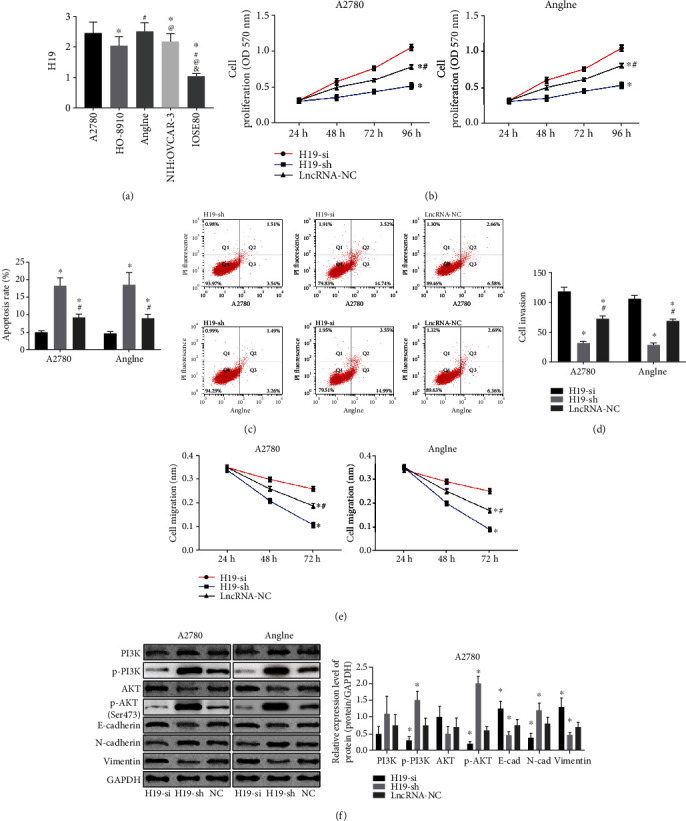
Effects of H19 on OC cells. (a) H19 expression level in OC cells and normal epithelial ovarian cells, ^∗^ represents *P* < 0.050 compared with A2780; ^#^ represents *P* < 0.050 compared with HO-8910; ^@^ represents *P* < 0.050 compared with Anglne; ^&^ represents *P* < 0.050 compared with NIH : OVCAR-3. (b) Proliferation of A2780 and Anglne cells after H19 transfection. (c) Apoptosis rate and flow cytometry of A2780 and Anglne cells after H19 transfection. (d) Invasion of A2780 and Anglne cells after H19 transfection. (e) Migration of A2780 and Anglne cells after H19 transfection. (f) Western blotting of A2780 and Anglne cells and relative expression levels of proteins in A2780 cells after H19 transfection. ^∗^ represents *P* < 0.050 compared with the H19-sh group; ^#^ represents *P* < 0.050 compared with the H19-si group.

Detection of H19 expression level in OC cell lines including A2780, HO-8910, Anglne and NIH : OVCAR-3, and normal ovarian epithelial cell line IOSE80 found that H19 was highly expressed in OC cells (*P* < 0.050) (Figure 2(a)), of which A2780 and Anglne cells had the highest expression levels (*P* < 0.050), so these two was chosen for subsequent experiments. H19 overexpression (H19-sh) and knockdown (H19-si) vectors were transfected into A2780 and Anglne cells, and the cytological behavior revealed that overexpression of H19 increased the ability of proliferation, invasion, and migration while decreased the apoptosis rate of OC cells (Figures 2(b)–2(e)). Moreover, western blot revealed that the expression of EMT marker proteins E-cadherin and Vimentin were increased, and N-cadherin was decreased when overexpression H19, indicating enhanced EMT (Figure 2(f)). The expression of p-PI3K and p-AKT were increased when upregulating H19 (*P* < 0.050), suggesting the activation of the PI3K/AKT pathway under H19 overexpression (Figure 2(f)). On the contrary, the inhibition of H19 expression resulted in the reversed effects.

### 3.3. Identification and Verification of the Correlation between H19 and miR-140-5p

**Figure 3:**
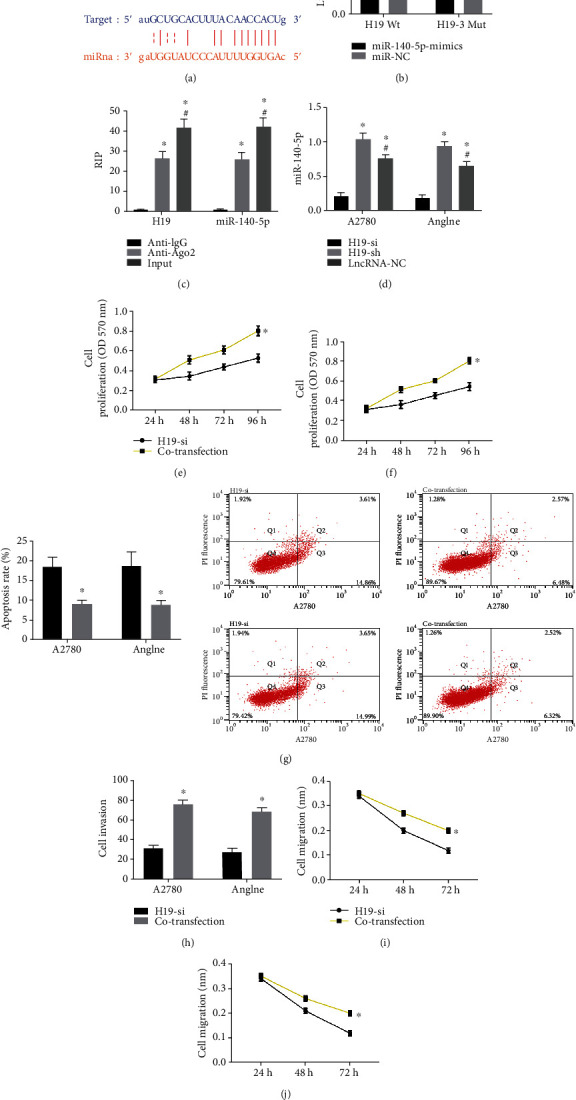
Validation of the relationship between H19 and miR-140-5p. (a) Predicted binding site. (b) DLR validated the targeting relationship between H19 and miR-140-5p. (c) RIP experiment. (d) MiR-140-5p expression level in A2780 and Anglne cells after transfection with H19, ^∗^ represents the comparison with the H19-sh group, *P* < 0.050; ^#^ stands for the comparison with the H19-si group, *P* < 0.050. (e) Proliferation of A2780 cells after cotransfection of H19-si and miR-inhibitor. (f) Proliferation of Anglne cells after cotransfection of H19 and miR-140-5p. (g) Apoptosis rate and flow cytometry of A2780 and Anglne cells after cotransfection of H19 and miR-140-5p. (h) Invasion of A2780 and Anglne cells after cotransfection of H19 and miR-140-5p. (i) Migration of A2780 cells after cotransfection of H19 and miR-140-5p. (j) Migration of Anglne cells after cotransfection of H19 and miR-140-5p. ^∗^ represents *P* < 0.050 compared with the H19-si group.

Through online website prediction, we found that there was a potential binding site of miR-140-5p on H19 (Figure 3(a)). Further dual-luciferase reporter assay demonstrated that miR-140-5p-mimics dramatically suppressed the fluorescence activity of H19-WT (*P* < 0.050) (Figure 3(b)). The levels of H19 and miR-140-5p precipitated by the Ago2 antibody were significantly higher than those of IgG (*P* < 0.050) (Figure 3(c)). After H19 was transfected into A2780 and Anglne cells, the expression level of miR-140-5p was detected. It was noticed that the miR-140-5p level in the H19 overexpression group was lower than that in the other two groups, while that in the H19 knockdown group was the highest (*P* < 0.050) (Figure 3(d)). The biological behavior of OC cells transfected merely with H19-si, and those cotransfected with H19-si and miR-140-5p inhibitor (cotransfection) were examined. It was found that cotransfection enhanced proliferation, invasion, and migration (Figures 3(e), 3(f), and 3(h)–(j)); inhibited cell apoptosis (Figure 3(g)); and promoted EMT compared to H19-si-transfected cells (Figures 3(k) and 3(l)).

### 3.4. Effects of miR-140-5p on OC Cells

**Figure 4:**
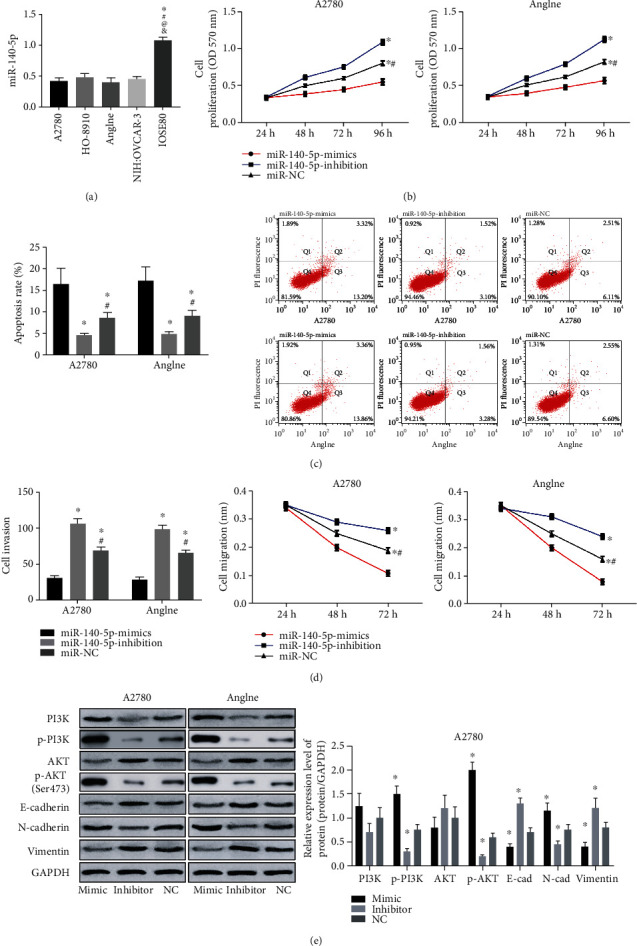
Effects of miR-140-5p on OC cells. (a) MiR-140-5p expression level in OC cells and normal ovarian epithelial cells, ^∗^ represents *P* < 0.050 compared with A2780; ^#^ represents *P* < 0.050 compared with HO-8910; ^@^ represents *P* < 0.050 compared with Anglne; ^&^ represents *P* < 0.050 compared with NIH : OVCAR-3. (b) Proliferation of A2780 and Anglne cells after miR-140-5p transfection. (c) Apoptosis rate and flow cytometry of A2780 and Anglne cells after miR-140-5p transfection. (d) Invasion of A2780 and Anglne cells after miR-140-5p transfection. (e) Migration of A2780 and Anglne cells after miR-140-5p transfection. Western blotting of A2780 and Anglne cells and relative expression levels of proteins in A2780 cells after miR-140-5p transfection. ^∗^ represents *P* < 0.050 compared with miR-140-5p-mimics group; ^#^ represents *P* < 0.050 compared with the miR-140-5p-inhibition group.

Detection of miR-140-5p expression levels in A2780, HO-8910, Anglne, NIH : OVCAR-3, and IOSE80 exhibited that miR-140-5p was lowly expressed in OC cells (*P* < 0.050) (Figure 4(a)). Then, miR-140-5p mimics (miR-mimics) and inhibitor (miR-inhibitor) were transfected into A2780 and Anglne cells, and the cytological behavior of the cells was detected to show that miR-140-5p mimics inhibited proliferation, invasion, and migration (Figures 4(b), 4(d), and 4(e)); increased cell apoptosis (Figure 4(c)); suppressed EMT; and downregulated PI3K/AKT pathway. In contrast, miR-140-5p inhibitor resulted in the reversed cytological behaviors.

### 3.5. H19 Overexpression Activated the PI3K/AKT/mTOR Pathway and the Downstream Protein Expression in Anglne Cells

**Figure 5:**
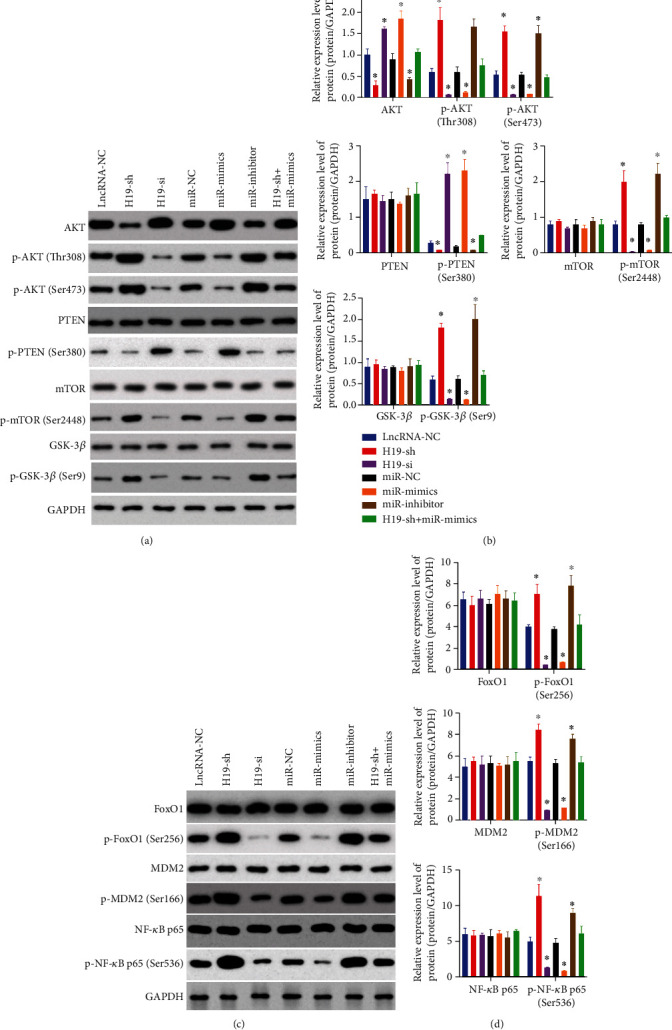
H19 overexpression activated the PI3K/AKT/mTOR pathway and the downstream protein expression in Anglne cells. (a, b) Western blot analysis of the PI3K/AKT/mTOR signaling pathway-related proteins in Anglne cells after tranfection. (c, d) Expression of the downstream proteins Forkhead box protein O1 (FoxO1), p-FoxO1 (Ser256), Murine Double Mimute 2 (MDM2), p-MDM2 (Ser166), NF-*κ*B p65, and p-NF-*κ*B p65 (Ser536) in Anglne cells after transfection was detected by western blot. All data were represented as mean ± S.D. ^∗^*P* < 0.05, *n* = 3 − 4.

We investigated the in vitro effect of H19 on the PI3K/AKT/mTOR signaling pathway and downstream protein expression by modulate the expression of H19 and miR-140-5p through transfection. As shown in Figure 5, western blot analysis demonstrated that with the overexpression of H19 or inhibition of miR-140-5p, the level of p-PTEN was decreased, while the expression of p-AKT (Thr308), p-AKT (Ser473), p-mTOR (Ser2448), p-FoxO1 (Ser256), p-MDM2 (Ser166), p-NF-*κ*B p65 (Ser536), and p-GSK-3*β* (Ser9) were markedly increased. No significant changes were observed in the expression of PTEN, GSK-3*β*, mTOR, FoxO1, MDM2, and NF-*κ*B p65 after overexpression or downregulation of H19 or miR-150-5p.

### 3.6. Tumorigenesis Experiment in Nude Mice

**Figure 6:**
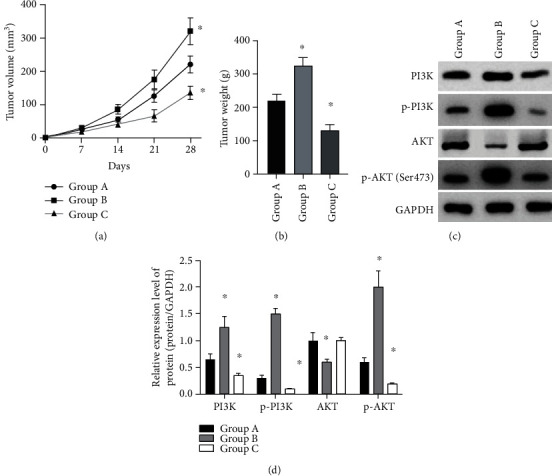
Tumorigenesis experiment in nude mice. (a) Changes of subcutaneous tumor volume in nude mice within 28 days. (b) Tumor volume at on day 28. (c, d) Relative expression and Western blotting of PI3K and AKT proteins in tumor formation. ^∗^*P* < 0.05.

When lncRNA-NC (group A), H19-sh (group B), and H19-sh+PI3K-inhibition (group C) were subcutaneously injected into the nude mice, the tumor volume and mass of nude mice in group B increased greatly (*P* < 0.050) (Figures 6(a) and 6(b)), and the tumor volume and mass in group C were lower than group A (*P* > 0.05). The expression of PI3K, phosphorylated PI3K (p-PI3K), and p-AKT protein in tumor tissue of nude mice from group B increased markedly (*P* < 0.05) (Figure 6(c)). As to group C, the level of p-PI3K and p-AKT protein decreased notably (*P* < 0.050).

## 4. Discussion

Ovarian cancer (OC) is currently the most lethal malignant tumor in female patients. As there are no significant special symptoms in the early stage, it is easy to be ignored or mishandled by patients. As a result, most patients have already reached the middle and late stages once diagnosed, at which time, most of the tumors present metastasis and invasion, greatly increasing the treatment difficulty and hazarding the prognosis of patients [20, 21]. Due to the high incidence and mortality of OC, it has become an important subject in the gynecological clinic that is in urgent need of a breakthrough [22]. With the deepening of research, molecular biology provides a new direction for modern tumor-targeting research [23, 24]. Previous evidence revealed that H19 and miR-140-5p are abnormally expressed and may affect the EMT of ovarian cancer cells. In this study, the in-depth study of the relation of H19 and miR-140-5p and the mechanism of action in OC is of great significance for the future clinical diagnosis and treatment of OC.

Our data showed that H19 was highly expressed while miR-140-5p was lowly expressed in the serum of OC patients, confirmed that H19 and miR-140-5p may act on the occurrence and development of OC. Based on ROC curve analysis, we found that H19 and miR-140-5p have good diagnostic efficacy in predicting the occurrence of OC, indicating that they may be effective serum markers for the diagnosis of OC in the future. Compared with the blood markers commonly used in clinics at present (such as CEA, CA199), H19 and miR-140-5p have more specific advantages, which is of great significance to help early identify the occurrence of OC. Through the correlation analysis, we found that there is a negative relation between H19 and miR-140-5p, suggesting that there is a certain correlation between them.

To further study the relationship between H19 and miR-140-5p on OC, we analyzed the expression of H19 and miR-140-5p in several ovarian cancer cell lines. The results showed that H19 was highly expressed in OC cells and miR-140-5p was lowly expressed, which confirmed the results of OC patients. However, by inhibiting the expression of H19, the proliferation, invasion, apoptosis, migration, and EMT of OC cells dropped, while the apoptosis rate increased. It is indicative that H19 plays a role of oncogenic gene in OC, which is in line with the study by Wu et al. on the mechanism of H19's effect on colorectal cancer [25], further supporting our results. While, overexpressed miR-140-5p elevated the proliferation, invasion, apoptosis, migration, and EMT of OC cells and reduced the apoptosis rate, suggesting its role as a tumor suppressor gene in OC, which was also confirmed by the results of Zhou et al. [26]. Similarly, H19 was shown to interact with miR-140-5p in lung cancer [27]. Therefore, in order to verify the relationship between the two, we predicted the binding site between H19 and miR-140-5p and confirmed the direct binding by dual-luciferase reporter assay and coimmunoprecipitation.

PI3K/AKT pathway is an important tumorigenesis pathway [28], which has been reported in research that the effect of miR-140-5p in renal cell carcinoma is closely related to PI3K/AKT [29]. Moreover, through tumorigenesis experiments in nude mice, we found that the increased activity of the PI3K/AKT pathway could promote tumor growth. We analyzed the activation of the PI3K/AKT pathway and downstream proteins by western blot and found that overexpression of H19 activated the PI3K/AKT and downstream proteins, as depicted by the upregulation of p-AKT, p-mTOR, and downregulation of p-PTEN. Therefore, our data suggested that H19 is implicated in the occurrence and development of OC by competing miR-140-5p and activating the PI3K/AKT signaling pathway. We confirmed the in vitro data by performing the tumorigenesis experiment in nude mice and found that overexpression H19 promoted tumor growth while PI3K inhibition restricted tumor growth. Recently, a TGF-beta/PD-L 1 bispecific antibody (YM101) was developed to treat cancer, which could reverse EMT in cancer [30]. It would be interesting to test whether YM101 could modulate the expression of H19 or miR-140-5p in treating OC.

To sum up, LncNA H19 competes the binding of miR-140-5p to activate the PI3K/AKT signal pathway to promote OC proliferation, invasion, migration, and EMT. Due to the experimental design, there are still some deficiencies in our study. The short experimental period resulted in the difficulty in evaluating the effect of H19 and miR-140-5p on the long-term prognosis of patients with OC. How miR-140-5p regulates PI3K/AKT pathway is not explored in this study. In addition, in the absence of experimental support, we are not sure whether the regulation of H19 on miR-140-5p has an impact on the efficacy of drug therapy for OC. In the future, we will focus on the targeted treatment of OC with H19 and further analyze the clinical value of H19.

## Figures and Tables

**Table 1:** Primer sequences.

	Upstream	Downstream
H19	5′-TTCAAAGCCTCCACGACTCT-3′	5′-GCTCACACTCACGCACACTC-3′
GAPDH	5′-CGGAGTCAACGGATTTGGTCGTAT-3′	5′-AGCCTTCTCCATGGTGGT-GAAGAC-3′
miR-140-5p	5′-CAGTGGTTTTACCCTATGG-TAGG-3′	5′-CGTGGTTCTACCCTGTGGTAG-3′
U6	5′-CTCGCTTCGGCAGCACA-3′	5′-AACGCTTCACGAATTTGCGT-3′

## Data Availability

All data generated or analyzed during this study are included in this published article.
